# Impact of Bounded Noise and Rewiring on the Formation and Instability of Spiral Waves in a Small-World Network of Hodgkin-Huxley Neurons

**DOI:** 10.1371/journal.pone.0171273

**Published:** 2017-01-27

**Authors:** Yuangen Yao, Haiyou Deng, Chengzhang Ma, Ming Yi, Jun Ma

**Affiliations:** 1 Department of Physics, College of Science, Huazhong Agricultural University, Wuhan, China; 2 Institute of Applied Physics, Huazhong Agricultural University, Wuhan, China; 3 Department of Physics, Lanzhou University of Technology, Lanzhou, China; 4 NAAM-Research Group, Department of Mathematics, Faculty of Science, King Abdulaziz University, Jeddah, Saudi Arabia; Universidad Rey Juan Carlos, SPAIN

## Abstract

Spiral waves are observed in the chemical, physical and biological systems, and the emergence of spiral waves in cardiac tissue is linked to some diseases such as heart ventricular fibrillation and epilepsy; thus it has importance in theoretical studies and potential medical applications. Noise is inevitable in neuronal systems and can change the electrical activities of neuron in different ways. Many previous theoretical studies about the impacts of noise on spiral waves focus an unbounded Gaussian noise and even colored noise. In this paper, the impacts of bounded noise and rewiring of network on the formation and instability of spiral waves are discussed in small-world (SW) network of Hodgkin-Huxley (HH) neurons through numerical simulations, and possible statistical analysis will be carried out. Firstly, we present SW network of HH neurons subjected to bounded noise. Then, it is numerically demonstrated that bounded noise with proper intensity *σ*, amplitude *A*, or frequency *f* can facilitate the formation of spiral waves when rewiring probability *p* is below certain thresholds. In other words, bounded noise-induced resonant behavior can occur in the SW network of neurons. In addition, rewiring probability *p* always impairs spiral waves, while spiral waves are confirmed to be robust for small *p*, thus shortcut-induced phase transition of spiral wave with the increase of *p* is induced. Furthermore, statistical factors of synchronization are calculated to discern the phase transition of spatial pattern, and it is confirmed that larger factor of synchronization is approached with increasing of rewiring probability *p*, and the stability of spiral wave is destroyed.

## Introduction

The neuronal system plays a pivotal role in regulating physiological behaviors [1–3], which consists of a large number of neurons. For instance, a neuron in the vertebrate cortex may link to as many as 10,000 postsynaptic neurons by synapses, which in turn can result in the formation of complex networks with complex topology [4, 5]. Recent studies have suggested that neuronal networks may share small-world (SW) topologies [6, 7], and neuronal dynamics can present and form complex spatial and temporal patterns [6]. However, it remains unclear how spatiotemporal patterns map biological functions. Spiral waves are one of the most important and typical spatiotemporal patterns, and have been observed in many biological systems, such as retinal spreading depression [8, 9], fertilizing Xenopus oocyte calcium waves [10], heart ventricular fibrillation [11], mammalian neocortex [12]. It is reported that spiral waves in the cardiac tissue could be very harmful, and the instability of spiral waves can result in ventricular fibrillation, even rapid death of heart. Therefore, removal and suppression of spiral waves in the cardiac tissue are in favor of preventing ventricular fibrillation. Moreover, spiral waves in the neocortex provide a spatial framework to organize cortical oscillations, thus help signal communication by coordinating oscillation phases over a group of neurons [12]. It is believed that spiral waves can regulate the collective behaviors like a pacemaker in the network, and the potential formation mechanism for spiral waves was discussed in a regular network [13–15]. In pathological conditions, spiral waves in the neocortex may extend the duration of evoked activity and interact with incoming signals, thus also may contribute to seizure generation [12]. Understanding of formation, instability and dynamics of spiral waves in biological systems can aid the dissecting of their biological functions. Furthermore, the spiral waves are widely observed in nonlinear systems involving physics, chemistry, and biology [16–18]. Therefore, the related studies about spiral waves are also beneficial to a better understanding of nonlinear dynamics.

Noise is inevitable in neuronal systems, and significantly influences the dynamics of nonlinear systems in many situations. It has been confirmed that noise can actually play a constructive role in many nonlinear systems. For instance, noise can amplify and optimize generally weak periodic input signal via the mechanism of stochastic resonance (SR) [19], and noise can enhance the regularity of spike activity in nonlinear systems via coherence resonance (CR) mechanism [20]. It is believed that noise can decrease the spatial regularity of network, for example, noise can induced breakup and instability of regular wave profiles [21, 22]. Aside from these two rather significant phenomena, many examples about noise-induced transitions [23], noise-induced transport [24], noise-induced synchronization [25], noise-sustained patterns [26], etc. are widely observed in diverse fields of studies ranging from physics and chemistry to biology. According to the different classified methods, noise could be treated as Gaussian white noise, colored white noise, Lévy noise, channel noise, etc [13]. Channel noise plays an important role in changing the membrane potential of single neuron and CR occurs to generate regular spiking in electrical activities when appropriate intensity of channel noise is considered [27]. Moreover, channel noise also can change the collective behaviors of neuronal network [28, 29]. Our studies have shown that channel noise often results in the breakup of ordered waves (spiral waves and target waves) in the neuronal network only optimized noise intensity can enhance stability and formation of spiral waves [30, 31]. Most of the previous works on the noise-induced phenomena subjected to Gaussian white or colored noise for convenience of analysis. Nevertheless, Gaussian noise has a chance of taking very large values, which violates the fact that the real physical quantity is always bounded [32]. In certain situation, non-Gaussian noises, such as bounded noise, phase noise, etc. could be more suitable for modeling realistic random processes [32]. Furthermore, some studies, especially experimental researches in sensory and biological systems [33], support the necessity of using non-Gaussian noises [34]. With the exception of Gaussian noise investigated in most previous studies, phenomena induced by other types of noise have also been widely observed and explored. For example, non-Gaussian noise can induce phase transition [34]. Phase noise can induce resonance in neuronal systems [35, 36]. Recently, bounded noise has aroused wide concern. And some interesting consequences have been obtained. For instance, bounded noise also can induce CR in neuronal systems [37, 38], transitions [39, 40], etc. Notwithstanding, the impacts of bounded noise on spiral waves in a SW neuronal network have not been reported for readers’ interests and guidance.

Human brain networks may share SW topology in connection [1, 6, 7]. A SW network presents clear clustered structure and sparsely long-range random connectivity [41], which not only can locally specialized work in sub-network composed of highly clustered nodes, but also can be globally integrated work in a highly efficient network with shortcuts [1]. This network architecture likely can maximize the complexity or adaption of function which it can support while also minimizing costs [7]. Noise is inherent to the nervous system. In realistic neuronal network, the connection between neurons could be time-varying. Therefore, the effects of noise and rewiring probability of SW network on spatiotemporal patterns and dynamics in neuronal systems attract much attention. Spatiotemporal dynamics in a SW network can be transformed remarkably when increasing the rewiring probability [42], and the rewiring probability can be used to successfully control spiral waves and spiral turbulence [42]. Our previous studies also found that noise with enough intensity can break spiral waves, while spiral waves are robust to appropriate noise. Meanwhile, moderate noise can facilitate formation and development of spiral waves [43, 44]. Furthermore, higher rewiring probability of SW network always destructs the spiral waves to spiral turbulence [43]. However, the concerned noise in most of studies is assumed to Gaussian noise, which is unbound, or the network is set as regular lattice. Recently, Yang *et al*. explored the impact of bounded noise and shortcuts on the spatiotemporal dynamics of SW neuronal networks, and founded that noise always impair spatial synchronization among coupled neurons, and yet CR occurs at an appropriately noise. Moreover, shortcuts in SW networks can enhance spatial synchronization and temporal coherence of network [5]. Motivated by the above results, investigating the impacts of bounded noise and shortcuts on spiral waves in a SW neuronal network is very interesting and important.

To our knowledge, impacts of bounded noise and shortcuts on spiral waves in a SW network have not been investigated. In this study, we mainly investigate roles of bounded noise and shortcuts on formation of spiral waves in a SW network of Hodgkin-Huxley (HH) neurons.

## Model

The spatiotemporal dynamics of HH neurons in a SW network is governed by the following differential equations [44].

$$C_{m}\frac{\text{d}V_{ij}}{\text{d}t}=g_{K}n_{ij}^{4}\left(V_{K}-V_{ij}\right)+g_{Na}m_{ij}^{3}h_{ij}\left(V_{Na}-V_{ij}\right)+g_{L}\left(V_{L}-V_{ij}\right)+D\sum_{kl}\varepsilon_{klij}\left(V_{kl}-V_{ij}\right)+\zeta$$ (1a)

$$\frac{\text{d}y_{ij}}{\text{d}t}=\alpha_{y}\left(V_{ij}\right)\left(1-y_{ij}\right)-\beta_{y}\left(V_{ij}\right)y_{ij},\;y=m,h,n$$ (1b)

$$\alpha_{m}=\frac{0.1(V_{ij}+40)}{1-\text{exp}(\text{−}(V_{ij}+40)/10)}$$ (2a)

$$\beta_{m}=4\text{exp}(\text{−}(V_{ij}+65)/18)$$ (2b)

$$\alpha_{h}=0.07\text{exp}(\text{−}(V_{ij}+65)/20)$$ (2c)

$$\beta_{h}=\frac{1}{1+\text{exp}(\text{−}(V_{ij}+35)/10)}$$ (2d)

$$\alpha_{n}=\frac{0.01(V_{ij}+55)}{1-\text{exp}(\text{−}(V_{ij}+55)/10)}$$ (2e)

$$\beta_{n}=0.125\text{exp}(\text{−}(V_{ij}+65)/80)$$ (2f)

where *V*_*ij*_ is the voltage of the cellular membrane of the neuron at the node (*i*, *j*), while *m*_*ij*_, *n*_*ij*_ and *h*_*ij*_ are parameters for gate channels of the neuron at the node (*i*, *j*). And ε_*ijkl*_ denote whether there is edge between two nodes. If the node (*k*, *l*) is connected to the node (*i*, *j*), then ε_*klij*_ *=* 1; otherwise, ε_*klij*_ *=* 0. According to previous procedure [45, 46], the studied networks are constructed at the beginning of each particular simulation, and then remain unchanged through the whole time for this particular simulation. Clearly, higher *p* of rewiring probability always means more shortcuts, while *p* = 0 and 1 correspond to the square lattice and a random regular graph, respectively. The membrane capacitance is *C*_*m*_ = 1 (μF/cm^2^). The maximal conductance constants for sodium, potassium, and leakage current is *g*_*Na*_ = 120, *g*_*K*_ = 36, and *g*_*L*_ = 0.3 (mS/cm^2^), respectively. And the reversal potentials for sodium, potassium, and leakage current is *V*_*Na*_ = 50, *V*_*K*_ = −77, and *V*_*L*_ = −54.4 (mV), respectively. Spiral waves can be induced and developed by many schemes. Here specific initial values with wedge-shaped type of *V*(41:43, 1:50) = −40.2, *V*(44:46, 1:50) = 0, *V*(47:49, 1:50) = 40.0, *m*(41:43, 1:50) = 0.1203, *m*(44:46, 1:50) = 0.5203, *m*(47:49, 1:50) = 0.98203, *h*(41:43, 1:50) = 0.9, *h*(44:46, 1:50) = 0.7, *h*(47:49, 1:50) = 0.5, *n*(41:43, 1:50) = 0.9, *n*(44:46, 1:50) = 0.7, *n*(47:49, 1:50) = 0.5 can be used to trigger a spiral seed, and then the perfect spiral wave can be induced and developed to occupy the network with the appropriate excitability [47]. And initial values for other neurons are selected with *V*(*i*, *j*) = −61.19389, *m*(*i*, *j*) = 0.08203, *h*(*i*, *j*) = 0.46012, *n*(*i*, *j*) = 0.37726. The coupling coefficient is set as *D* = 0.5, time step Δ*t* = 0.001 ms, neurons number *N* × *N* = 100 × 100, the Euler forward difference procedure and no-flux boundary condition are adopted in our numerical simulations.

In the formula of (1a), *ζ* represents bounded noise, which is described as follows [5, 37]:

ζ(t)=Asin(ωt+σW(t)) (3)

Where the noise amplitude *A* ≥ 0; *ω* (= 2π*f*) stands for angular frequency. *W* (*t*) is the standard Wiener process, while *σ* denotes the intensity of the unit Wiener process *W* (*t*). For *t* → ∞, the mean, autocorrelation function, and power spectral density of bounded noise are obtained by [5, 37]:

〈ζ(t)〉=0 (4)

$$\langle \zeta \left(t\right)\zeta \left(t+\tau \right)\rangle =\frac{A^{2}}{2}\text{exp}\left(-\frac{\sigma^{2}\tau }{2}\right)\text{cos}\left(\omega \tau \right),\;\tau >0$$ (5)

$$S\left(\omega^{\prime }\right)=\frac{{\left(A\sigma \right)}^{2}}{2\pi }\left[\frac{1}{4{\left(\omega -\omega^{\prime }\right)}^{2}+\sigma^{4}}+\frac{1}{4{\left(\omega +\omega^{\prime }\right)}^{2}+\sigma^{4}}\right]$$ (6)

Indeed, the power spectrum of bounded noise depends on the parameters of *A*, *ω* and *σ* synchronously, which presents two symmetrical peaks at *ω*′ = ±*ω*. The bandwidth of bounded noise is mainly dominated by *σ*. For *σ* → +∞, white noise is approached, while it corresponds to narrow-band process for small enough *σ*. Especially, the bounded noise turns into a sinusoidal periodic signal when *σ =* 0. As previously mentioned, the time evolution of a standard Wiener process is generated in our numerical simulations by the following formula [40]:

$$W\left(t\right)=W\left(t-\Delta t\right)+\sqrt{-2\Delta t\text{ln}\chi_{1}}\text{cos}\left(2\pi \chi_{2}\right)$$ (7)

where Δ*t* indicates time step, and *χ*_1_, *χ*_2_ are two independent random numbers between 0 and 1 with equal probability [40]. The initial value of 0.3 is adopted for *W* in our numerical simulations.

Based on the mean-field theory, synchronization factor *R* is used to characterize spatial synchronization [43, 44] and pattern selection.

$$F=\frac{1}{N^{2}}\sum_{j=1}^{N}\sum_{i=1}^{N}V_{ij}$$ (8a)

$$R=\frac{\langle F^{2}\rangle -{\langle F\rangle }^{2}}{\frac{1}{N^{2}}\sum_{j=1}^{N}\sum_{i=1}^{N}\left(\langle V_{ij}^{2}\rangle -{\langle V_{ij}\rangle }^{2}\right)}$$ (8b)

where *V*_*ij*_ is the membrane potential of the neuron at the node (*i*, *j*), while *N*^2^ stands for the total number of all neurons. And the symbol of <*> denotes averaging over time. Obviously, larger *R* denotes better synchronization and is harmful for survival of regular waves. Moreover, *R* approaches 0 and 1 that corresponds to no and perfect synchronization, respectively. The sudden change of curve of *R* vs. bifurcation parameter can indicates the phase transition [43, 44]. A sufficiently large time window of 500 time units is used in the calculation of *R*. By the way, extensive numerical results with the different initial values can find similar statistical conclusions and pattern selections.

## Main results

**Fig 1:**
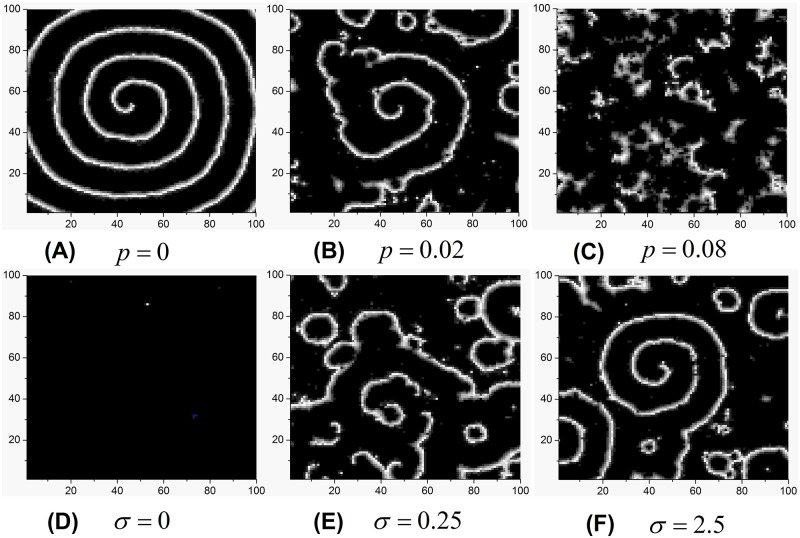
Developed patterns in the SW network at *t* = 500 time units, which are obtained by fixing *A* = 10, *f* = 80 and adjusting *p* and *σ*, respectively. *σ* = 1 is adopted in top panel. *p* = 0.02 is adopted in bottom panel.

Firstly, we discuss how rewiring probability *p* and intensity σ influence the wave formation and instability of spiral waves. For regular network i.e. *p* = 0, the spiral wave can be induced (Fig 1A). With increase of rewiring probability *p*, the developed spiral patterns will be destroyed (Fig 1B). By further increasing *p* and it is found that spiral waves are removed completely (Fig 1C). Therefore, higher rewiring probability *p* usually destroys spiral waves by introducing the shortcuts in the regular network that the network can become heterogeneous and/or synchronous.

**Fig 2:**
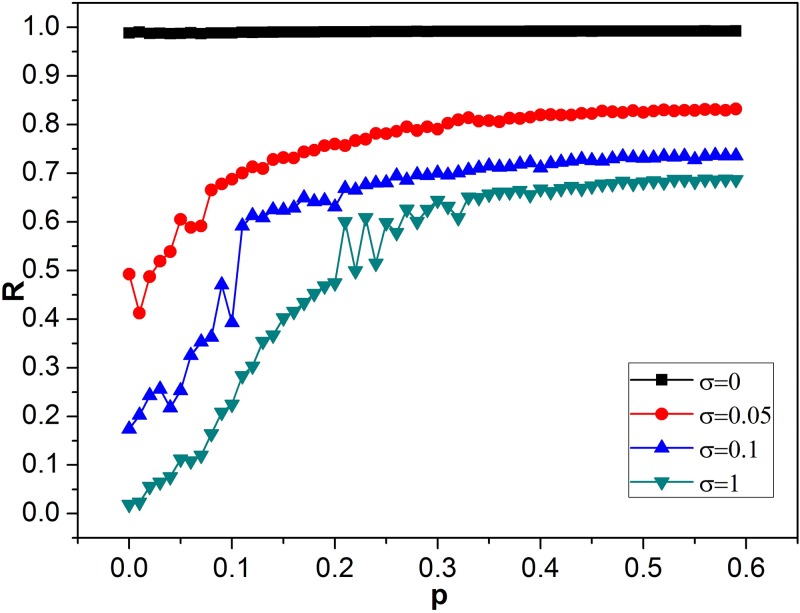
The synchronization factor *R* as a function of rewiring probability *p* for the different intensity *σ*. And *A* = 10 and *f* = 80 are adopted.

**Fig 3:**
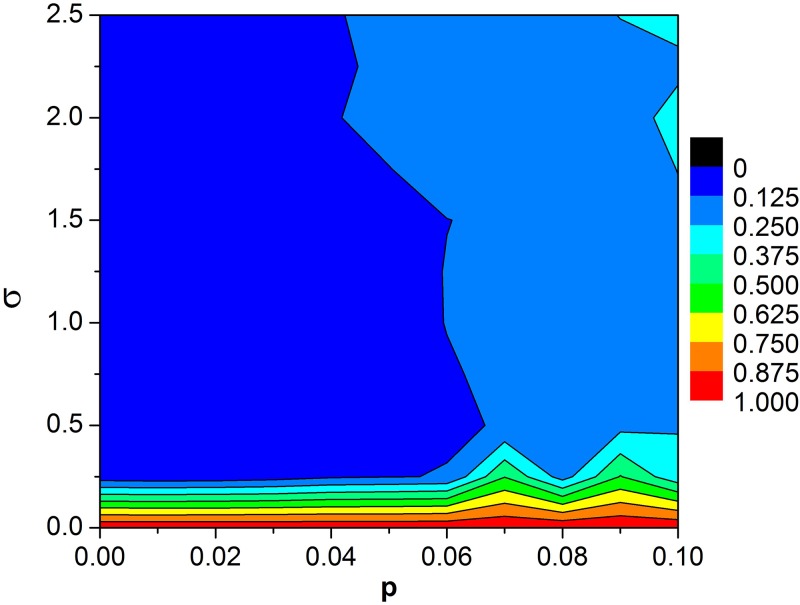
Contour plot of *R* in the *σ—p* plane. Other parameters for bounded noise are set to *A* = 10 and *f* = 80.

Then, the influence of intensity *σ* of the unit Wiener process on the spiral waves in the SW network is investigated by fixing a *p* = 0.02. For *σ* = 0, all neurons have not been fired (Fig 1D). But spiral wave appears gradually for the increasing *σ* (Fig 1E). One can observe the formation of spiral waves clearly when *σ* is large enough (Fig 1F). Namely, even in SW network, such as *p* = 0.02, *σ* can facilitate the formation of spiral waves. To further confirm the above observed phenomena quantitatively, the synchronization factor of *R* vs. increasing *p* is plotted for the different order parameter *σ* (Fig 2). For *σ* = 0, *R* is close to 1, and almost keeps unchanged with the increasing of *p* (Fig 2), which corresponds to nearly perfect synchronization. As a whole, as *p* is increased, *R* ascends monotonously for *σ* > 0. But the ascent rate of *R* gradually reduces to nearly zero, and thus a “platform” arises finally during *p* proceeds (Fig 2). Moreover, a sudden change of *R—p* curve appears at 0.05 for *σ* = 0.05, 0.1 for *σ* = 0.1, or 0.2 for *σ* = 1, which indicates the phase transition of patterns (Fig 2). On the whole, the increasing *σ* results in the reduction of *R*, and smaller factor of synchronization *R* indicates high possibility to induce spiral waves. Therefore, *σ* can induce the formation of spiral waves, which is consistent with the results of Fig 1. The smaller *p* is, the more obvious this induction effect of *σ* is (Fig 2). Consequently, it is very interesting to search the range of parameters of *p* and *σ*, for which the spiral wave can survive and occupy in the network. As shown in contour plot of *R* in the *σ—p* plane (Fig 3), spiral waves keep robust when the rewiring probability *p* is below certain values about 0.04 ~ 0.06. Moreover, this result also confirms that the larger *σ* is, the more easily it induces the formation of spiral waves from the vertical point of view (Fig 3). However, the induction effect of *σ* reaches saturation when *σ* continuously is increased (Fig 3). Although spiral waves can keep robust in the SW network with small *p*, there is shortcut-induced phase transition of spiral wave from formation to breakup with the increase of rewiring probability *p* from the horizontal point of view (Fig 3).

**Fig 4:**
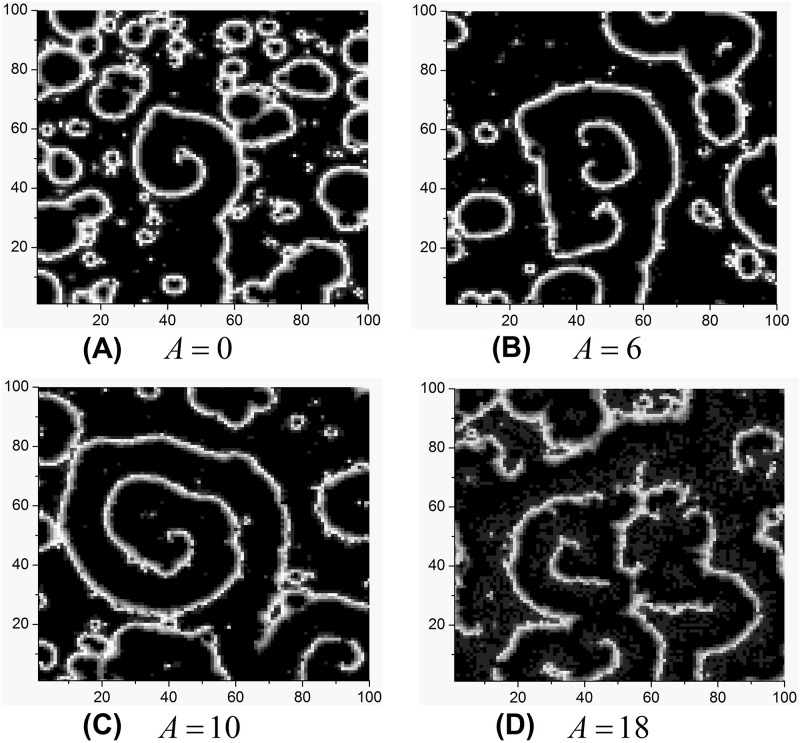
Developed patterns in the SW network at *t* = 500 time units, which are obtained by fixing *f* = 80, *σ* = 1, *p* = 0.02 and adjusting amplitude *A*.

**Fig 5:**
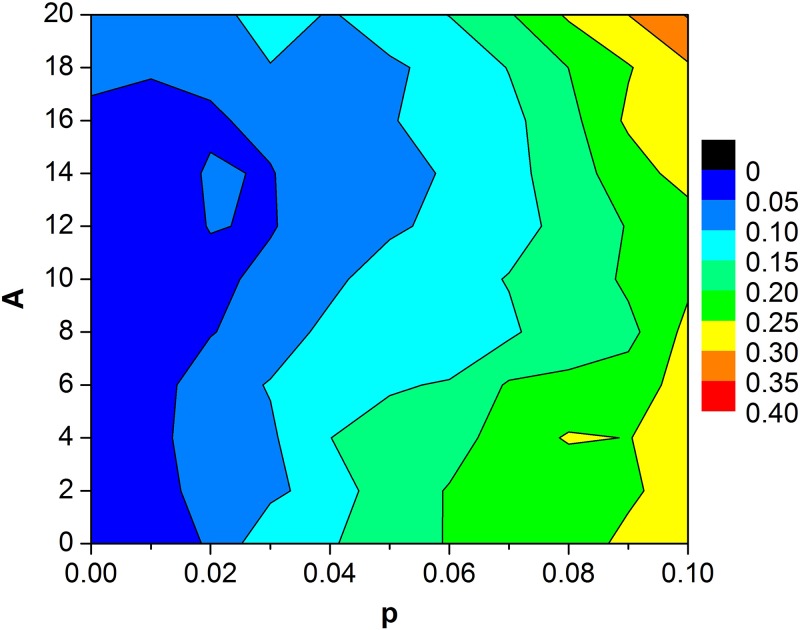
Contour plot of *R* in the *A—p* plane. Other parameters for bounded noise are set to *σ* = 1 and *f* = 80.

**Fig 6:**
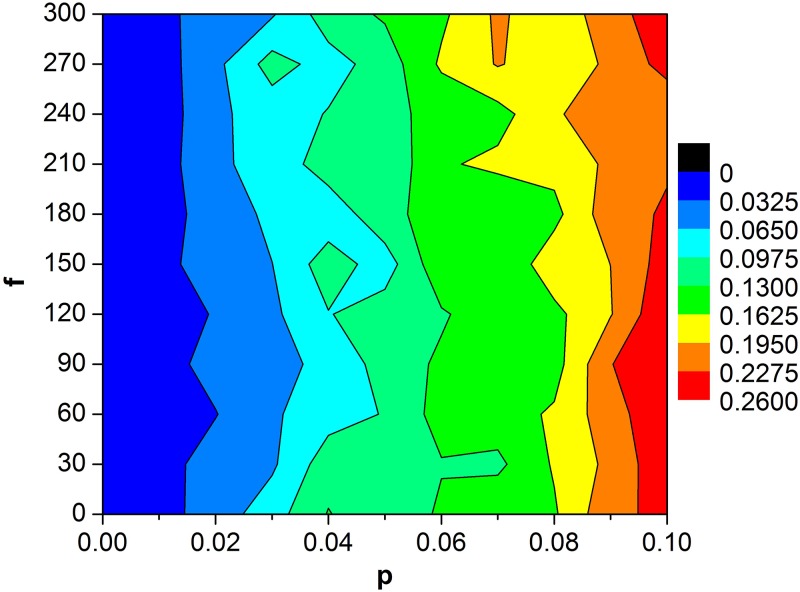
Contour plot of *R* in the *f—p* plane. Other parameters for bounded noise are set to *A* = 10 and *σ* = 1.

In what follows, we present the influences of amplitude and frequency of bounded noise on pattern formation and destruction of spiral waves. Some developed spiral waves are shown in Fig 4 when *p* = 0.02 is fixed. It is found that moderate amplitude can facilitate the formation of spiral wave (Fig 4). To further get a global view, the contour plot of *R* in the *A*—*p* plane is depicted in Fig 5 when we fix *f* = 80 and *σ* = 1. The dark and light blue regions in the left indicate the appearance of spiral wave (Fig 5). In addition, the *R* varies with *A* when *p* is fixed, such as *p* = 0.02, the plot of *R* versus *A* presents a resonant behavior. Namely, there exists optimal amplitude, which facilitates the formation of spiral waves. It is suggested that spiral waves can be controlled by regulating the amplitude of bounded noise in SW networks of HH neurons (Fig 5). The similar phenomenon also can be observed with appropriate parameters setting, such as *p* = 0.04 when the frequency of bounded noise is carefully changed, which is illustrated in Fig 6.

## Conclusion

As previously described in the section of introduction, as a reasonable and random excitation model, bounded noise has been widely applied in many fields. However, few related results about bonded noise on spiral waves in a SW neuronal network have been presented until today. The present study focuses bounded noise and shortcuts on selection of spiral waves in SW networks of HH Neurons. Through numerical simulations, it can be observed that bounded noise with proper intensity *σ*, amplitude *A*, or frequency *f* can facilitate the formation of spiral waves when rewiring probability *p* is below certain values. And resonant behavior can occur in the SW networks. In addition, rewiring probability *p* always impairs spiral waves. Although spiral waves can keep robust in the SW networks with small *p*, with the increase of *p*, the shortcut-induced destruction of spiral waves in SW networks of HH neurons can be observed. Taken together, the presented results not only make for investigating the effect of bounded noise on the spiral waves in the SW nervous system, but also lay foundations for related researches involving bounded noise.

## Supporting information

S1 File The data for contour plot of R in the *σ-p* plane (Fig 3). (TXT)

S2 File The data for contour plot of R in the *A-p* plane (Fig 5). (TXT)

S3 File The data for contour plot of R in the *f-p* plane (Fig 6). (TXT)
